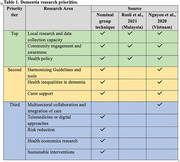# Dementia Research Priorities in Low‐ and Middle‐income Countries in Southeast Asia: A scoping review of the literature and nominal group technique outcome

**DOI:** 10.1002/alz.086022

**Published:** 2025-01-09

**Authors:** Siti Maisarah Mattap, Devi Mohan, Hoon Jia Xi, Dinesh Sangarran Ramachandram, Yuda Turana, Maw Pin Tan, Tuan Anh Nguyen, Gloria HY Wong, Veeda Michelle M. Anlacan, DY Suharya, Susan Moloney, Louise Robinson

**Affiliations:** ^1^ Jeffrey Cheah School of Medicine and Health Sciences, Monash University Malaysia, Bandar Sunway Malaysia; ^2^ School of Public Health, The University of Queensland, Brisbane, QLD Australia; ^3^ School of Pharmacy, Monash University Malaysia, Bandar Sunway Malaysia; ^4^ Atma Jaya Catholic University of Indonesia, Jakarta Indonesia; ^5^ University of Malaya, Kuala Lumpur Malaysia; ^6^ Swinburne University of Technology, Hawthorn, VIC Australia; ^7^ National Ageing Research Institute, Melbourne, VIC Australia; ^8^ The University of Hong Kong, Hong Kong, Hong Kong Hong Kong; ^9^ College of Medicine and Philippine General Hospital, University of the Philippines Manila, Manila Philippines; ^10^ Alzheimer’s Disease International for the Asia Pacific Region, Alzheimer’s Disease International, London United Kingdom; ^11^ Population Health Sciences Institute, Newcastle University, Newcastle upon Tyne United Kingdom; ^12^ Newcastle University, Newcastle upon Tyne United Kingdom

## Abstract

**Background:**

Dementia is a global public health concern, that poses daily challenges to the individuals, families, and healthcare systems worldwide. Sixty percent of those affected reside in low‐ and middle‐income countries (LMICs), where 71% of new cases are anticipated by 2025. Most dementia studies focus on high‐income countries, emphasizing the need for region‐specific investigations in areas like Southeast Asia, where diverse cultural, economic, and healthcare settings present unique complexities. Addressing specific challenges and priorities in Southeast Asia will facilitate tailored interventions and effective strategies. We aim to map dementia research priorities in Southeast Asian LMICs and align them with stakeholder‐identified priorities through a consultation process using the nominal group technique.

**Method:**

A scoping review was conducted utilizing the Joanna Briggs Institute guidance. Four databases (OvidMedline, Scopus, PsycINFO, and CINAHL) were searched for eligible studies reporting dementia research priorities in LMICs in Southeast Asian. Comparisons were made to a stakeholders’ consultation during a two‐day workshop from the 9^th^ to 10^th^ February 2023 in Kuala Lumpur, Malaysia. Participants included the Southeast Asia‐Dementia (SEA‐Dem) Research Network members key stakeholders from Malaysia, Indonesia, Vietnam, Philippines, Singapore, and Hong Kong (n = 20). Research priorities from each participating country were generated and ranked, harmonized with those from the nominal group technique into tiers of priorities.

**Result:**

Only two studies from Malaysia and Vietnam were eligible, reporting unranked research priorities. Nominal group technique ranked outcomes from Malaysia, Vietnam, Indonesia, and the Philippines were included. Top dementia research priorities were (1) local research and data collection capacity, (2) community awareness and engagement, and (3) health policy. Second‐tier research priorities included harmonizing guidelines and tools standardization, health inequalities, and availability of carer support. The third tier comprised multisectoral collaboration, integration of care, telemedicine, digital approaches, dementia risk reduction, health economics, and sustainable interventions.

**Conclusion:**

Our ranked and harmonized latest dementia research priorities list can serve as a more nuanced and contextually informed dementia research directional guide for countries with similar backgrounds. Collaborative efforts to increase high‐quality dementia research capacity in Southeast Asian LMICs should be intensified for better dementia care in the region.